# Deletion of the *H240R* Gene of African Swine Fever Virus Decreases Infectious Progeny Virus Production Due to Aberrant Virion Morphogenesis and Enhances Inflammatory Cytokine Expression in Porcine Macrophages

**DOI:** 10.1128/jvi.01667-21

**Published:** 2022-02-09

**Authors:** Pingping Zhou, Lian-Feng Li, Kehui Zhang, Bing Wang, Lijie Tang, Miao Li, Tao Wang, Yuan Sun, Su Li, Hua-Ji Qiu

**Affiliations:** a State Key Laboratory of Veterinary Biotechnology, National African Swine Fever Para-reference Laboratory, National High Containment Facilities for Animal Diseases Control and Prevention, Harbin Veterinary Research Institute, Chinese Academy of Agricultural Sciences, Harbin, China; b Northeast Agricultural University, Harbin, China; University of North Carolina at Chapel Hill

**Keywords:** African swine fever virus, H240R protein, infectious progeny virions, virus assembly, inflammatory cytokines

## Abstract

African swine fever virus (ASFV) is a complex nucleocytoplasmic large DNA virus that causes African swine fever, a lethal hemorrhagic disease that currently threatens the pig industry. Recent studies have identified the viral structural proteins of infectious ASFV particles. However, the functional roles of several ASFV structural proteins remain largely unknown. Here, we characterized the function of the ASFV structural protein H240R (pH240R) in virus morphogenesis. pH240R was identified as a capsid protein by using immunoelectron microscopy and interacted with the major capsid protein p72 by pulldown assays. Using a recombinant ASFV, ASFV-ΔH240R, with the *H240R* gene deleted from the wild-type ASFV (ASFV-WT) genome, we revealed that the infectious progeny virus titers were reduced by approximately 2.0 logs compared with those of ASFV-WT. Furthermore, we demonstrated that the growth defect was due to the generation of noninfectious particles with a higher particle-to-infectious titer ratio in ASFV-ΔH240R-infected primary porcine alveolar macrophages (PAMs) than in those infected with ASFV-WT. Importantly, we found that pH240R did not affect virus-cell binding, endocytosis, or egress but did affect ASFV assembly; noninfectious virions containing large aberrant tubular and bilobulate structures comprised nearly 98% of all virions observed in ASFV-ΔH240R-infected PAMs by electron microscopy. Notably, we demonstrated that ASFV-ΔH240R infection induced high-level expression of inflammatory cytokines in PAMs. Collectively, we show for the first time that pH240R is essential for ASFV icosahedral capsid formation and infectious particle production. Also, these results highlight the importance of pH240R in ASFV morphogenesis and provide a novel target for the development of ASF vaccines and antivirals.

**IMPORTANCE** African swine fever is a lethal hemorrhagic disease of global concern that is caused by African swine fever virus (ASFV). Despite extensive research, there exist relevant gaps in knowledge of the fundamental biology of the viral life cycle. In this study, we identified pH240R as a capsid protein that interacts with the major capsid protein p72. Furthermore, we showed that pH240R was required for the efficient production of infectious progeny virions as indicated by the *H240R-*deleted ASFV mutant (ASFV-ΔH240R). More specifically, pH240R directs the morphogenesis of ASFV toward the icosahedral capsid in the process of assembly. In addition, ASFV-ΔH240R infection induced high-level expression of inflammatory cytokines in primary porcine alveolar macrophages. Our results elucidate the role of pH240R in the process of ASFV assembly, which may instruct future research on effective vaccines or antiviral strategies.

## INTRODUCTION

African swine fever (ASF) is a devastating disease to the pig industry worldwide caused by African swine fever virus (ASFV). No commercial vaccine or antiviral strategy is currently available for the disease. ASFV is a member of the *Asfarviridae* family belonging to the group of nucleocytoplasmic large DNA viruses (NCLDVs) and the only known DNA arbovirus (1, 2). ASFV is a large enveloped double-stranded DNA (dsDNA) virus containing a linear DNA genome of 170 to 190 kb that encodes more than 150 open reading frames (ORFs) (3–8), depending on the strain. ASFV particles are 260 to 300 nm in diameter (9) and composed of complex multilayered structures with an overall icosahedral morphology despite sharing structural, genomic, and replicative characteristics with other NCLDVs (3). ASFV differs by possessing a multilayered structure and an overall icosahedral morphology. Intracellular ASFV has a genome-containing nucleoid surrounded by a thick protein layer referred to as the core shell, which is wrapped by an inner lipid envelope and an icosahedral capsid; these four layers are comprised of more than 50 proteins (10). Extracellular ASFV gains an external envelope (outer envelope) as it buds through the plasma membrane (11).

ASFV primarily targets and replicates in swine monocytes and macrophages, which are mainly present in the blood and bone marrow (12), and the infective cycle begins with virus entry into host cells by either clathrin-mediated endocytosis or macropinocytosis (13). After internalization, the virus particles undergo a low-pH-driven disassembly process, leading to a membrane fusion event between the inner envelope and the late endosomal membrane, ultimately releasing the genome-containing core into the cytoplasm (14). DNA replication and assembly of ASFV occur in the cytoplasm of infected cells in viral factories located close to the cell nucleus (15–17). ASFV assembly is a complex and dynamic process that requires a series of cooperative protein interactions. A mass spectrometry-based analysis of purified ASFV particles identified 68 virus-encoded and 21 host cellular proteins (10). ASFV encodes at least 16 of the 68 detected proteins involved in the assembly of the virus particle, as previously described (10). Intracellular virus particles are formed by the progressive assembly of the outer capsid, including one major and four minor capsid proteins, which are assembled into pentasymmetrons and trisymmetrons (4). Structural analyses using cryogenic electronic microscopy (cryo-EM) reconstruction have unveiled the basis of capsid stability and assembly (9, 10, 18), opening up new avenues for ASF vaccine development.

A previous study has revealed that several proteins, including p72, pB438L, and p17, are involved in the formation of the outer capsid (9). Analysis of the structure of ASFV by cryo-EM has shown that during the early stage of viral assembly, the interaction between minor capsid protein pB438L and the penton proteins mediates the docking of the penton complex to the inner membrane, where it recruits capsomers to form the penton core, initiating virus assembly (9). p72 is the major capsid protein (MCP) and constitutes most of the external capsid, whereas the penton proteins form the vertex (18). Minor capsid proteins, including p17 and M1249L, are located below the external capsid shell, presumably to stabilize the viral capsid (18). It has been demonstrated that the penton proteins play crucial roles in the process of ASFV assembly. Based on molecular weight estimation and secondary structure prediction, pH240R, a structural protein, is largely presumed to be a pentameric capsid protein designated the penton protein (9). In a proteomic analysis of extracellular ASFV particles, the hitherto uncharacterized viral protein pH240R was detected in abundance (10). To a large extent, capsid proteins may play important roles in the process of virus assembly and then affect the morphogenesis of the virus; thus, gene deletion in ASFV may result in a reduction in viral replication. As capsid proteins of ASFV, p72 and pB438L are essential for the formation of infectious virus particles (19, 20). The porcine adenovirus type 3 E3 region encodes the capsid protein 13.7K, which reduces viral replication by influencing the formation of stable capsids and production of infectious progeny virions (21). To the best of our knowledge, no data are currently available on the role of pH240R in the life cycle of ASFV.

In this study, the functional role of pH240R in the life cycle of ASFV was characterized. The results showed that pH240R was associated with ASFV morphogenesis, behaving as a capsid protein that interacted with p72 by pulldown assays. Using a recombinant ASFV with the *H240R* deletion (ASFV-ΔH240R), we found that pH240R was required for the efficient production of infectious progeny virions. Importantly, ASFV-ΔH240R failed to efficiently assemble after virus entry into the cells and generated 98% aberrant tubular and bilobulate virion structures, resulting in 1.7-log growth defect. In addition, ASFV-ΔH240R infection induced high-level inflammatory cytokine expression associated with the tumor necrosis factor (TNF) signaling pathway, including TNF-α, interleukin 1β (IL-1β), IL-6, C-X-C motif chemokine ligand 8 (CXCL8), and IL-10, in primary porcine alveolar macrophages (PAMs) compared with that induced by the wild-type ASFV HLJ/2018, resulting in partial growth defect. Altogether, our study identifies a new capsid protein of ASFV and provides a novel target for the development of anti-ASFV strategies that block the infectious cycle of ASFV.

## RESULTS

### Conservation of H240R protein in different ASFV isolates and transcription of *H240R* gene in the ASFV replication cycle.

**FIG 1:**
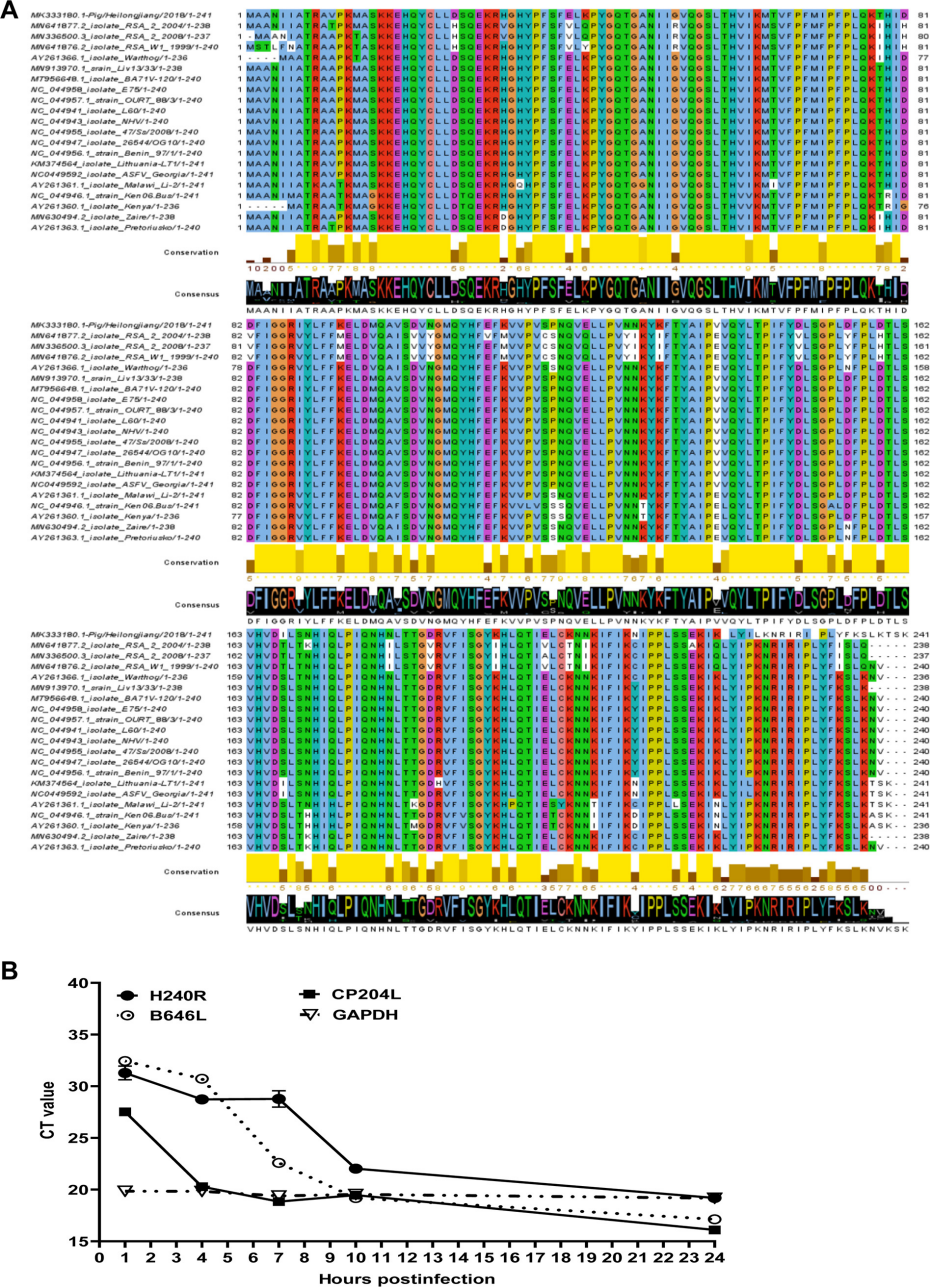
Biological characteristics of the H240R protein (pH240R). (A) Multiple sequence alignment of pH240R of 21 ASFV isolates. To assess the nature of the replacements at multiple residues, conservation scores based on the biological properties of each amino acid were included, with lower scores associated with more divergent replacements. Analysis was conducted on Jalview software version 2.11.1.4, using the ClustalW algorithm. (B) *H240R* gene transcriptional dynamics. Average cycle threshold values of the ASFV HLJ/2018-infected primary porcine alveolar macrophages (MOI of 5) at 1, 4, 7, 10, and 24 hpi were examined by quantitative reverse transcription-PCR analyses using primers against *H240R*, *CP204L*, *B646L*, and *GAPDH*.

The wild-type ASFV HLJ/2018 (ASFV-WT) *H240R* gene (*H240R*) is located on the forward strand between nucleotide positions 155339 and 156064 in the genome and encodes the H240R protein (pH240R) with 241 amino acids. Twenty-one ASFV isolates were examined by multiple sequence alignment using Jalview software version 2.11.1.4. The data showed a very high degree of amino acid sequence similarity, with 84.66% to 100.00% similarity among isolates containing the same or different forms of pH240R (Fig. 1A). Low levels of amino acid identity (84.66%) could be observed between isolate Malawi/Lil 20–1/1983 and isolates RSA_W1_199–4, Zaire-20, RSA_2_2004-20, and RSA_2_2008-22. In contrast, 98.28% conservation was observed with isolates L60, OURT_88/3, 47/Ss/2008, NHV, Benin_97/1, 26544/OG10, and E75. And 100% amino acid identity was found between isolate ASFV-WT and isolate Georgia 2007/1. Interestingly, no differences were found at amino acid levels within the Eurasian lineage isolates, indicating conservation of pH240R within this lineage.

To determine the transcription kinetics of *H240R*, total RNA was extracted from the PAMs infected with ASFV-WT at a multiplicity of infection (MOI) of 5. The transcription level of *H240R* was detected by using quantitative reverse transcription-PCR (qRT-PCR). As shown in Fig. 1B, the mRNA level of *CP204L* (p30) was increased rapidly between 1 and 4 h postinfection (hpi), reflected by decreasing cycle threshold (*C_T_*) values which exhibit early gene expression during ASFV infection. *H240R* transcription was decreased at 4 hpi but rapidly increased between 7 and 10 hpi, similar to the late transcriptional gene *B646L.* The results demonstrated that *H240R* is a late transcriptional gene of ASFV. It remains to be elucidated whether the low amounts of *B646L* and *H240R* mRNA detected at an early time are due to basal *de novo* transcription or remnants of the input material.

### Subcellular and subviral localization and interaction with p72 of pH240R.

**FIG 2:**
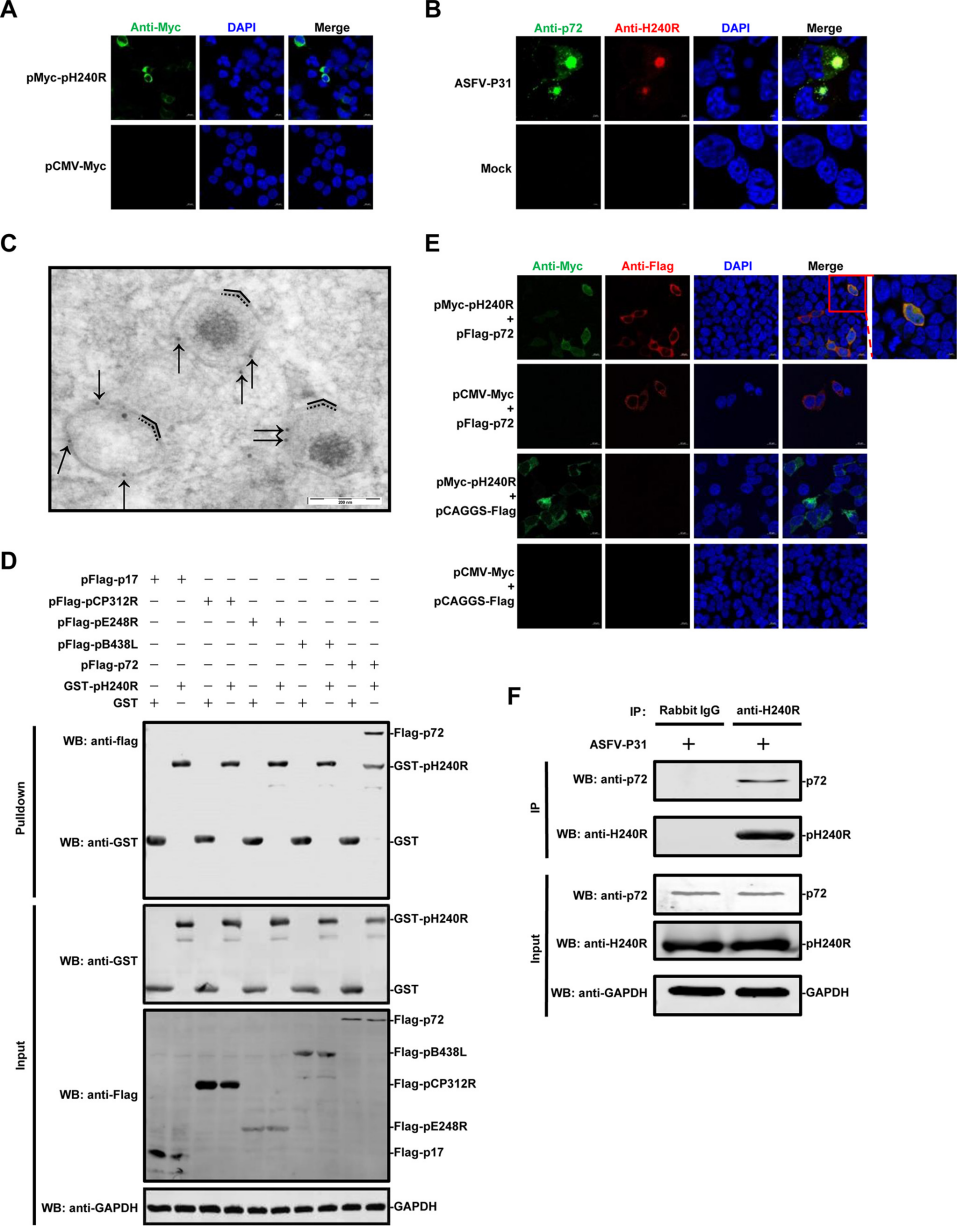
The H240R protein (pH240R) was identified as a capsid protein of ASFV. (A) Intracellular localization of pH240R. HEK293T cells were transfected with plasmid pMyc-pH240R (1 μg). After 24 hpi, the cells were fixed and probed with a mouse MAb and the nuclear marker DAPI and then detected by confocal microscopy. Bars, 10 μm. (B) Subcellular localization of pH240R. HEK293T cells infected with HEK293T-adapted ASFV (ASFV-P31) were immobilized at 18 hpi and immunolabelled with rabbit anti-pH240R pAb, anti-p72 Mab, and DAPI. Bars, 2 μm. (C) Subviral localization of pH240R. ASFV HLJ/2018-infected primary porcine alveolar macrophages were fixed at 18 hpi and immunolabeled with anti-pH240R PAb, followed by conjugation to 10-nm-diameter gold particles. The arrows indicate that gold particles presenting on intracellular viruses were associated with the outer protein capsid (solid lines) outside of the inner envelope (dotted lines). (D) Identification of the interaction between pH240R and p72. To screen the pH240R-interacting protein(s) related to ASFV morphogenesis, HEK293T cells were transfected with plasmids pFlag-p17, pFlag-pE248R, pFlag-pCP312R, pFlag-pB438L, and pFlag-p72 (6 μg each), respectively. GST pulldown and immunoblotting analyses were performed with anti-GST or anti-Flag MAb. WB, Western blotting. (E) Colocalization of pH240R with p72. HEK293T cells were transfected with pFlag-p72 (1 μg) alone or together with pMyc-pH240R (1 μg) plasmids and analyzed by immunofluorescence assay for pH240R, p72, and DAPI. Bars, 10 μm. (F) Endogenous co-IP assay. HEK293T cells were infected with ASFV-P31 (MOI = 1) for 36 h and subjected to a coimmunoprecipitation (co-IP) assay using anti-p72 MAb and anti-H240R PAb. Irrelevant rabbit IgG was used as a negative control.

Correspondingly, to investigate the subcellular localization of pH240R, HEK293T cells were transfected with plasmid pCMV-pH240R or control plasmid pCMV-Myc for 24 h. Confocal microscopy confirmed that Myc-tagged pH240R was localized in the cytoplasm (Fig. 2A).

We further explored the subcellular localization of pH240R in HEK293T cells infected with the HEK293T-adapted ASFV (ASFV-P31) (22) by confocal microscopy. As shown in Fig. 2B, the fluorescence of pH240R was detected mainly within the perinuclear viral factories, without punctate structures scattered throughout the cytoplasm, as suggested by its colocalization with the MCP p72. To investigate the precise localization in the virus particles of pH240R, immunoelectron microscopy (IEM) was performed on ultrathin cryosections of the ASFV-infected PAMs fixed at 18 hpi. The arrows indicate that gold particles were labeled on the external layer of the icosahedral virions, the capsid (Fig. 2C). Together, the results indicate that pH240R is a capsid component exposed on the surface of the intracellular virions.

Previous studies have shown that interactions between viral inner membrane and capsid proteins are required for ASFV capsid assembly (9, 18). To investigate the proteins that interact with pH240R in ASFV morphogenesis, inner membrane proteins (p17, pE248R, and pCP312R) and capsid proteins (pB438L and p72) were selected for validation by glutathione *S*-transferase (GST) pulldown assay and confocal microscopy. As shown in Fig. 2D, the GST-tagged pH240R (GST-pH240R) protein pulled down the Flag-tagged p72 (Flag-p72) protein. Consistent with the results, colocalization of pH240R and p72 was observed in the cytoplasm of HEK293T cells (Fig. 2E). To further evaluate whether the endogenous pH240R interacts with p72, HEK293T cells were infected with ASFV-P31 for a coimmunoprecipitation (co-IP) assay. As shown in Fig. 2F, endogenous pH240R was coimmunoprecipitated with p72 in the ASFV-P31-infected cells.

### pH240R affects the viral growth but not the hemadsorption of ASFV in PAMs.

**FIG 3:**
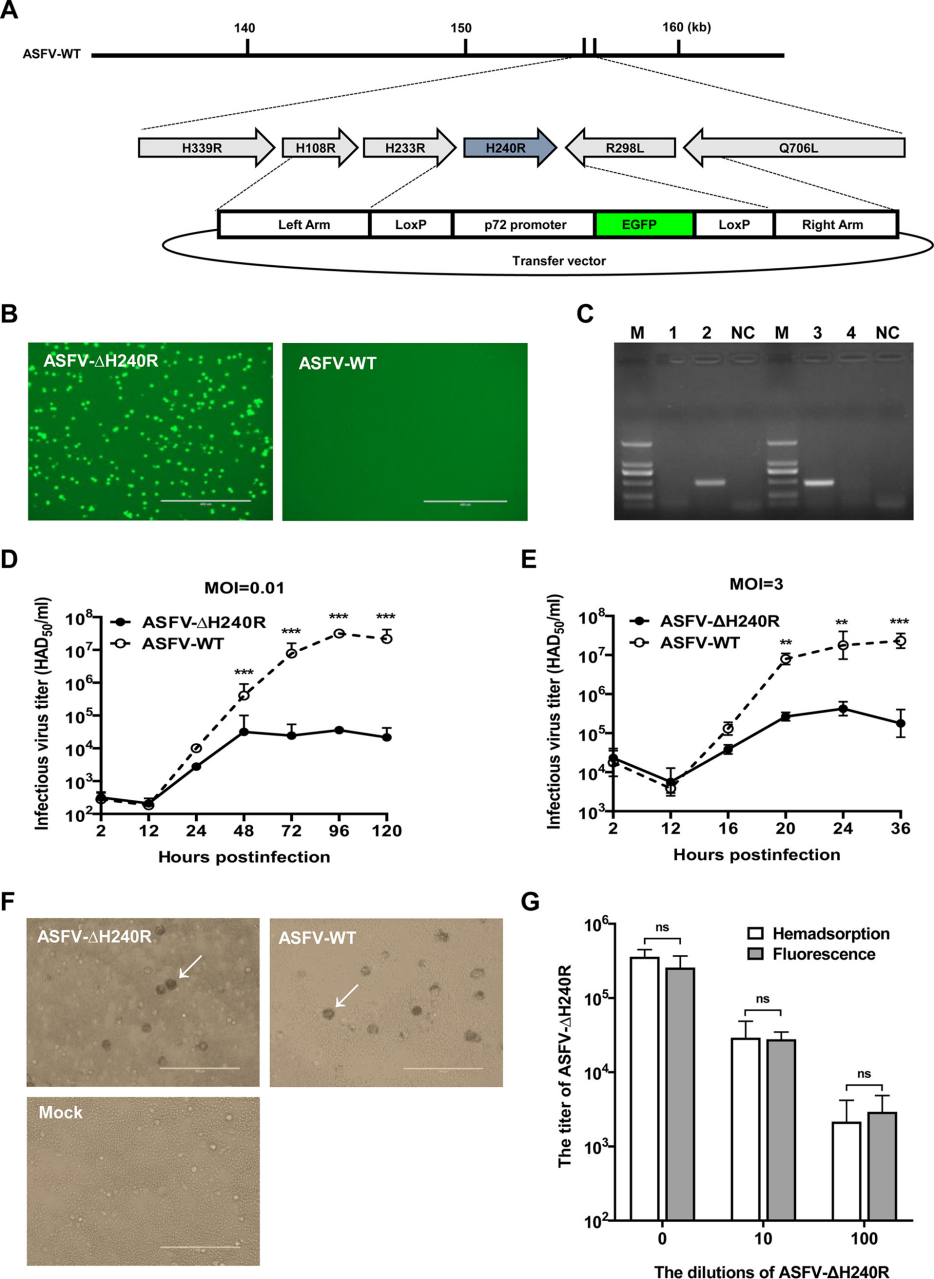
ASFV-ΔH240R showed growth defect but retained hemadsorption characteristics in primary porcine alveolar macrophages (PAMs). (A) Schematic diagram of the genome structure of the gene-deleted ASFV HLJ/2018 (ASFV-ΔH240R). The recombinant transfer vector containing the left arm, EGFP marker, and right arm was designed to sequentially delete *H240R* at nucleotides 155339 to 156064 of the ASFV-WT genome. ASFV-ΔH240R was finally purified by limiting dilution in 96-well plates based on the EGFP signal. (B) ASFV-ΔH240R was amplified in PAMs for downstream evaluation. (C) Identification of the *H240R* gene deletion in ASFV-ΔH240R. *H240R* was examined by PCR in both ASFV-ΔH240R (lane 1) and ASFV HLJ/2018 (ASFV-WT) (lane 3) using primers d/H240R-F/R, and the p72EGFP gene was examined by PCR in both ASFV-ΔH240R (lane 2) and ASFV-WT (lane 4) using primers 72EGFP-F/R. NC, negative control. (D and E) *In vitro* replication characteristics of ASFV-ΔH240R or ASFV-WT. PAMs were infected at an MOI of 0.01 (D) or 3 (E) with ASFV-ΔH240R or ASFV-WT. The cell cultures were collected at each time point, and growth curves were constructed by using the titer of each virus at each time point. The data represent the results of three independent experiments. Error bars denote standard errors of the means. The significance of differences between groups (*n *= 3) was determined using Student's *t* test (**, *P* < 0.01;***, *P < *0.001). (F) Hemadsorption characteristics of ASFV-ΔH240R. PAMs were infected with ASFV-ΔH240R or ASFV-WT, and uninfected cells were included as a negative control. Hemadsorption was detected under a microscope (TE2000-U; Nikon, Japan). The arrows indicated “rosettes” of red blood cells. (G) ASFV-ΔH240R titers determined by hemadsorption or fluorescence assays. PAMs were infected with ASFV-ΔH240R at 10-fold serial dilutions for 5 days. Hemadsorption and green fluorescence were detected under a microscope (TE2000-U Nikon, Japan). The data represent three independent experiments. Error bars denote standard errors of the means. The significance of differences between groups (*n* = 3) was determined using Student's *t* test. ns, not significant.

To investigate the role of *H240R* in ASFV infection in PAMs, a recombinant virus lacking *H240R*, ASFV-ΔH240R, was generated using a DNA homologous recombination approach as previously described (16). ASFV-ΔH240R bearing a cassette containing the enhanced green fluorescent protein (EGFP) gene under the control of the ASFV p72 promoter (p72EGFP) (Fig. 3A) was purified after 14 rounds of limiting dilution purification based on the fluorescence in PAMs and was amplified in PAMs to generate a virus stock (Fig. 3B).

To evaluate the purity of the recombinant virus stock, viral DNA was extracted from the ASFV-ΔH240R or ASFV-WT stocks and analyzed by PCR using primers targeting *H240R* (Table 1). Amplification for *H240R* was detected with DNA extracted from ASFV-WT, but not from ASFV-ΔH240R (Fig. 3C), indicating the lack of contamination of ASFV-ΔH240R with ASFV-WT. The DNA sequence assemblies of ASFV-ΔH240R revealed a gene deletion of 723 bp from the full *H240R* corresponding to the introduced modification. The consensus sequence of the ASFV-ΔH240R genome showed a gene insertion of 1,002 bp corresponding to the p72EGFP cassette sequence introduced without any nucleotides in *H240R*. Except for the insertion of the cassette, ASFV-ΔH240R did not result in any mutations during the process of homologous recombination or purification.

**TABLE 1:** Primers used in this study^*a*^

Primer	Sequence (5′–3′)	Description
Myc-H240R-F	CATTTTGGCAAAGAATTCATGGCTGCAAACATTATT	For the gene *H240R*
Myc-H240R-R	GCAGAGGGAAAAAGATCTGCTAGCTCGAGTTACA	
B646L-F	GAATTCGGATGGCATCAGGAGGAG	For the gene *B646L*
B646L-R	CTCGAGTTAGGTACTGTAACGCAGCA	
LA-F	GGAGCTCGAATTCGTCTAGTTATATATGTCGGTCA	For the left arm
LA-R	GACTTTTCTCCGGCGACCCTTTATGAACATATGTT	
72EGFP-F	AACATATGTTCATAAAGGGTCGCCGGAGAAAAGTC	For the marker gene
72EGFP-R	GGTTAAATAATTAATATATAGTTATCTAGATCCGGTG	
RA-F	CACCGGATCTAGATAACTATATATTAATTATTTAACC	For the right arm
RA-R	TCTGCAGAAGCTTCGAATTCGATATTTTGGCTATC	
d/H240R-F	GTCATGGGCATTATCCCTT	For parental virus
d/H240R-R	TTTGCAGGTGTTTATATCCAG	
r/p72EGFP-F	TCGTCCTAACCCTAAAGGC	For recombinant virus
r/p72EGFP-R	GCTTAAAATTATTGACACGCTCT	
H240R-F	ATGCAATACCACTTCGAG	qRT-PCR for H240R
H240R-R	CACTACCGGTATAGCATA	
B646L(p72)-F	CTGCTCATGGTATCAATCTTATCGA	qRT-PCR for p72
B646L(p72)-R	GATACCACAAGATC(AG)GCCGT	
CP204L(p30)-F	CGGTAGAATTGTTACGAC	qRT-PCR for CP204L
CP204L(p30)-R	TTCTTGAGCCTGATGTTC	
GAPDH-F	GAAGGTCGGAGTGAACGGATTT	qRT-PCR for GAPDH
GAPDH-R	TGGGTGGAATCATACTGGAACA	
TNF-α-F	CCTACTGCACTTCGAGGTTATC	qRT-PCR for TNF-α
TNF-α-R	ACGGGCTTATCTGAGGTTTG	
IL-1β-F	ACCCAAAACCTGGACCTTGG	qRT-PCR for IL-1β
IL-1β-R	CATCACAGAAGGCCTGGGAG	
IL-6-F	CTCATTAAGTACATCCTCGG	qRT-PCR for IL-6
IL-6-R	GTCTCCTGATTGAACCCAGA	
CXCL8-F	GCAACAACAACAGCAGTAAC	qRT-PCR for CXCL8
CXCL8-R	TGACCAGCACAGGAATGA	
IL-10-F	GCAGCCAGCATTAAGTCTGA	qRT-PCR for IL-10
IL-10-R	CTGGTCCTTCGTTTGAAAGA	
D117L-F	GCACGAACTGAGATATATCGT	For the gene *D117L*
D117L-R	CGTGGTGAACGTACTTGCCAT	
CP312R-F	ATGTTACTAGTAAAAATG	For the gene *CP312R*
CP312R-R	TT AAGCAATAGCAATCTG	
E248R-F	ATGGGAGGCTCTACAAGCAA	For the gene *E248R*
E248R-R	TTACGAAACGGCAGCATTTT	
B438L-F	ATGTATCATGATTATGCCTC	For the gene *B438L*
B438L-R	TTAAAGGGATGGTGATATGGA	

a LA, left arm; RA, right arm; d, deletion; r, recombinant; F, forward; R, reverse.

To examine the replication efficiency of ASFV-ΔH240R *in vitro*, PAMs were infected with ASFV-ΔH240R or ASFV-WT at the low MOI of 0.01 and the high MOI of 3 to evaluate whether the *H240R* deletion affected viral replication. The results showed that the growth kinetics of ASFV-ΔH240R were significantly reduced by approximately 2.0 logs compared with those of ASFV-WT depending on the time point considered (Fig. 3D and E), indicating that the *H240R* deletion significantly decreased the viral growth of ASFV-ΔH240R in PAMs. Hemadsorption characteristics were determined by adding porcine red blood cells to the virus-infected PAMs, and “rosettes” of red blood cells were observed on the ASFV-ΔH240R- or ASFV-WT-infected PAMs, demonstrating that the ASFV with *H240R* deletion still maintained the hemadsorption property of ASFV (Fig. 3F). To titrate ASFV-ΔH240R efficiently in later experiments, hemadsorption and fluorescence assay results were compared. The results demonstrated that there were no significant differences in the final titers determined by the two methods (Fig. 3G).

### Deletion of *H240R* results in decreased infectious progeny virus production but not viral genome replication.

**FIG 4:**
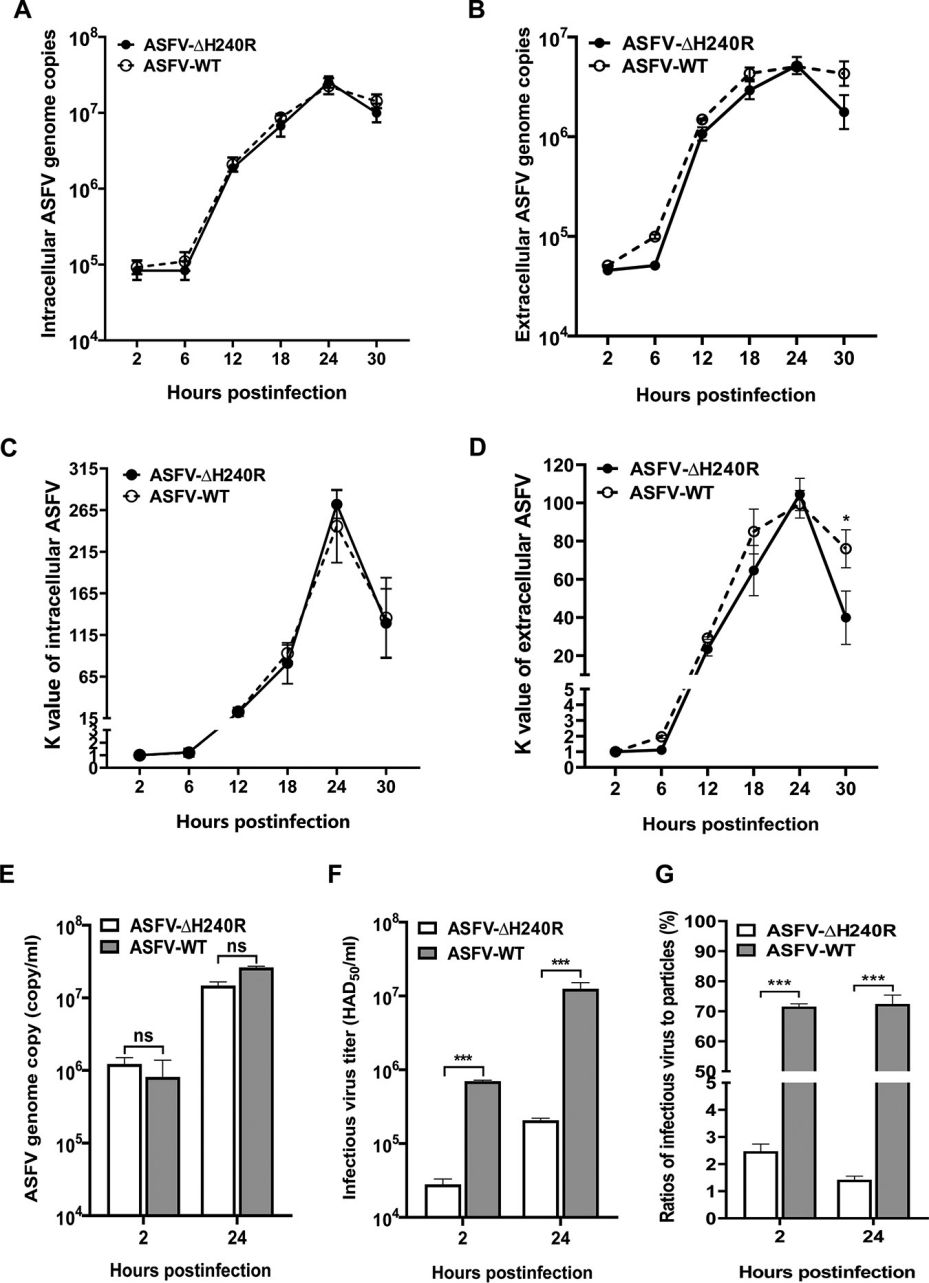
ASFV-ΔH240R shows decreased viral production but normal genome replication in primary porcine alveolar macrophages (PAMs). (A and B) Intracellular (A) and extracellular (B) genome copies of ASFV-ΔH240R or ASFV HLJ/2018 (ASFV-WT). PAMs in 24-well plates were infected with either ASFV-ΔH240R or ASFV-WT at an MOI of 3. At the indicated hpi, the numbers of genome copies in the cell lysates or supernatants of infected PAMs were determined by quantitative PCR (qPCR). (C and D) Dynamics of intracellular or extracellular ASFV replication. The intracellular (C) or extracellular (D) *K* value represents the proliferation curve of the virus at each time point and is calculated by dividing the number of virus genome copies at each time point by that at 2 h. The viral proliferation curves were generated using the virus genome copies at 2, 6, 12, 18, 24, and 30 hpi. (E) Genome copies of progeny ASFV-ΔH240R or ASFV-WT. PAMs in 24-well plates were infected with either ASFV-ΔH240R or ASFV-WT at genome copies of 10^7^. At the indicated hpi, the numbers of genome copies in supernatants were determined by qPCR. (F) Infectious progeny virus production of ASFV-ΔH240R or ASFV-WT. The titers of infectious progeny virions from panel E were detected based on the Reed and Muench calculation method. (G) ASFV-ΔH240R or ASFV-WT infectious virus-to-particle ratios. The infectious virus-to-particle ratios were calculated based on the ratio of the titers (E) to the genome copies (F) at 2 or 24 hpi (*n *= 3). The data shown are from three independent experiments. Error bars denote standard errors of the means. The significance of differences between groups (*n *= 3) was determined using Student's *t* test (*, *P < *0.05; ***, *P < *0.001). ns, not significant.

The growth kinetics showed that the *H240R* deletion resulted in the partial production of replication-competent virions but with 2.0-log-fewer infectious progeny virions than ASFV-WT. To investigate the effect of *H240R* deletion on viral genome replication, the cell lysates or supernatants from the PAMs infected with ASFV-ΔH240R or ASFV-WT at equal numbers of genome copies were harvested at different hpi, and the ASFV genome copies were quantified by quantitative PCR (qPCR). Interestingly, no significant differences were observed in the numbers of intracellular or extracellular ASFV genome copies (Fig. 4A and B) and the viral proliferation curves (Fig. 4C and D) at each time point, as both were similar to those observed for ASFV-WT. We then measured the ASFV genome copies and titers in the cellular supernatant collected at 2 and 24 hpi. Consistent with the previous data, no significant differences were observed in the numbers of ASFV genome copies among samples collected at various hpi (Fig. 4E). Intriguingly, infectious progeny virus production was significantly different in cell-free particles released from ASFV-ΔH240R- and ASFV-WT-infected PAMs (Fig. 4F). The infectious virus-to-particle ratios (titer/genome copy) for ASFV-ΔH240R were significantly lower than those for ASFV-WT. That is, only 1.4% progeny of ASFV-ΔH240R had the ability to successfully infect PAMs, while approximately 72.4% progeny of ASFV-WT had the ability to infect PAMs at 24 hpi (Fig. 4G), indicating that PAMs infected with ASFV-ΔH240R produced larger amounts of noninfectious particles.

### Deletion of *H240R* causes aberrant ASFV virion morphogenesis in PAMs.

**FIG 5:**
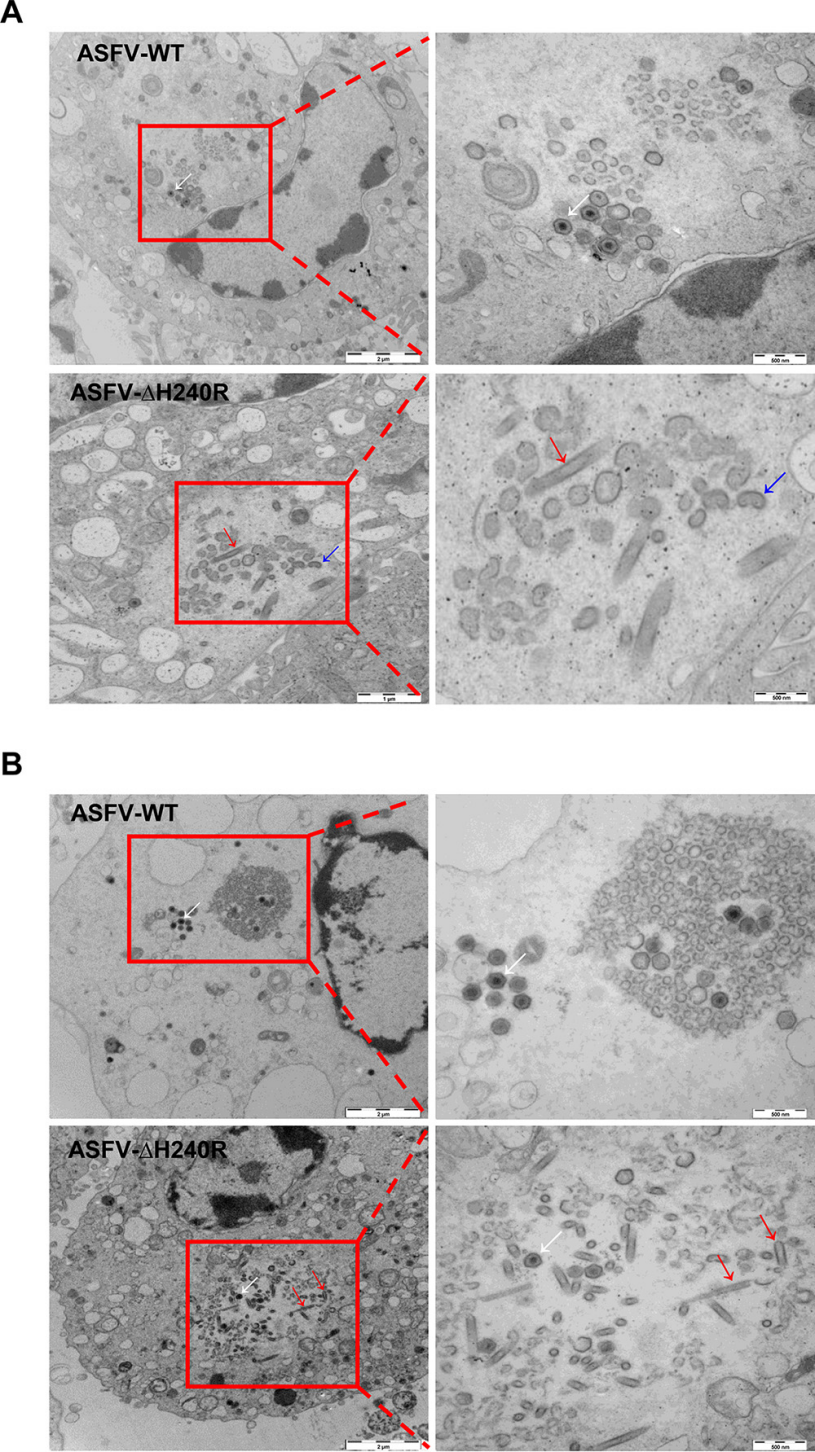
Deletion of the *H240R* gene from ASFV causes aberrant ASFV virion morphogenesis. (A) Transmission electron microscopy (TEM) analysis of ASFV HLJ/2018 (ASFV-WT)- or ASFV-ΔH240R-infected primary porcine alveolar macrophages (PAMs) at 24 hpi. The assembly sites contain amounts of precursor viral membranes, as well as immature and mature virions in ASFV-WT-infected PAMs (white arrows). The factories contain, in addition to precursor membranes as well as a small number of mature virions (white arrows), large numbers of aberrant tubular (red arrows) and bilobulate (blue arrows) structures in the ASFV-ΔH240R-infected PAMs. (B) Ultrathin sections of ASFV-WT- or ASFV-ΔH240R-infected PAMs are shown at 48 hpi. The morphologies of virions in the viral factories from PAMs infected with ASFV-WT or ASFV-ΔH240R are consistent with those at 24 hpi; the difference is that the number of normal structures in the ASFV-WT-infected PAMs (white arrows) or aberrant structures in ASFV-ΔH240R-infected PAMs (red arrows) increased.

As a capsid protein, pH240R may affect the assembly of ASFV and further alter viral replication efficiency. To verify the hypothesis that alteration in viral assembly can cause growth defect, we then performed an ultrastructural analysis of ASFV-ΔH240R- or ASFV-WT-infected PAMs at 24 and 48 h by transmission electron microscopy (TEM). As shown in Fig. 5A, the viral factories in the ASFV-WT-infected PAMs at 24 hpi consisted of abundant precursor viral membranes, immature viruses (IMVs) and mature viruses (MVs) (white arrows), and viruses containing empty capsids. Conversely, the assembly sites contained precursor viral membranes and only a few mature icosahedral particles, while numerous tubular structures (red arrows) with a length of 1 μm or more and bilobulate structures (blue arrows) were found in the viral factories in ASFV-ΔH240R-infected PAMs at 24 hpi. To determine whether the absence of pH240R from ASFV delayed viral assembly, TEM analysis was performed on PAMs infected with ASFV-ΔH240R or ASFV-WT at 48 hpi. As shown in Fig. 5B, the viral factories in the ASFV-WT-infected PAMs at 48 hpi were similar to those at 24 hpi but contained more precursor viral membranes as well as IMVs and MVs. Consistent with the results at 24 hpi, the viruses in the viral factories in ASFV-ΔH240R-infected PAMs at 48 hpi contained a few MVs and a large number of particles with tubular structures.

**FIG 6:**
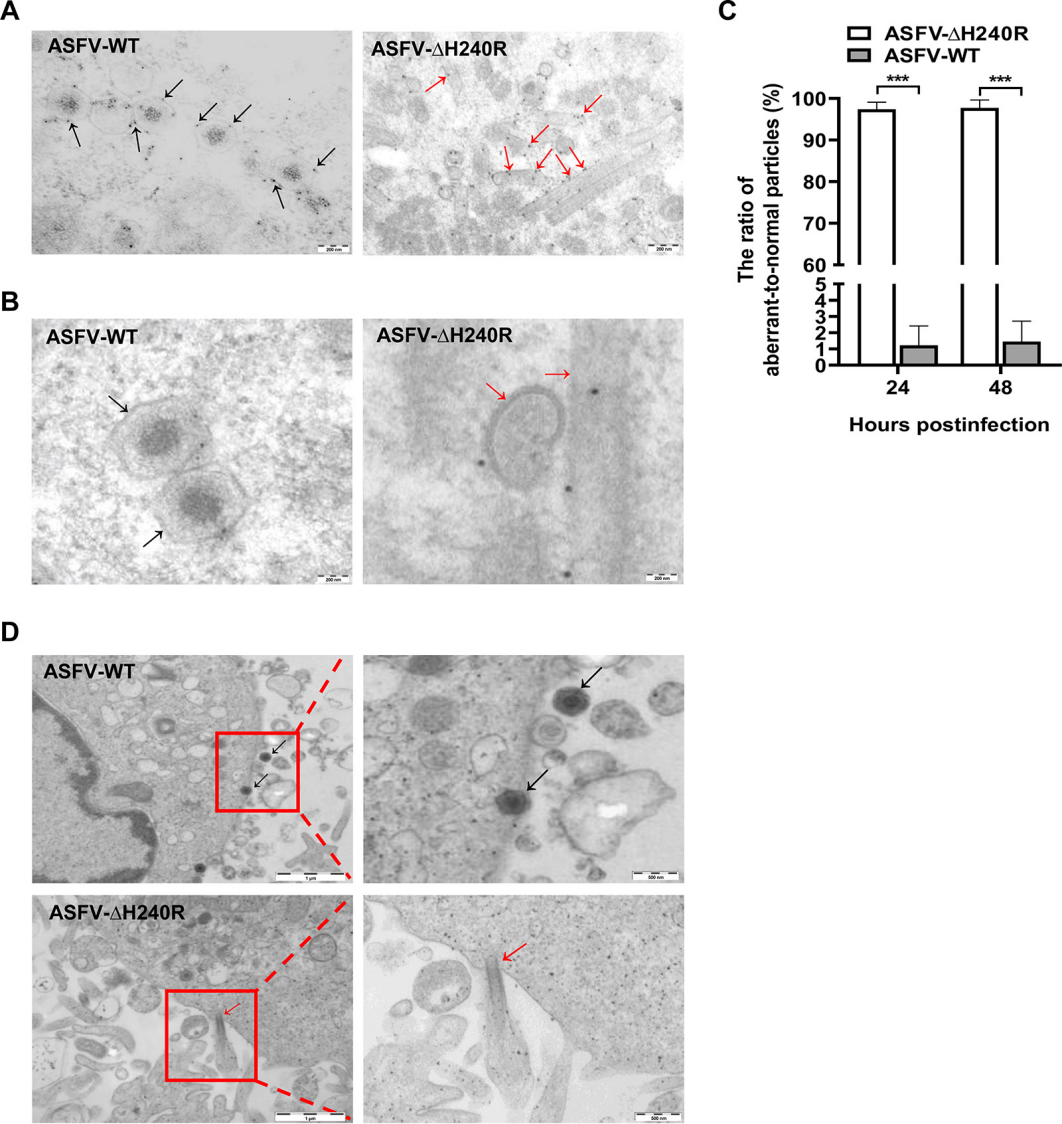
ASFV-ΔH240R displays aberrant virion structures. (A) Immunoelectron microscopy (IEM) analysis of ASFV HLJ/2018 (ASFV-WT)- or ASFV-ΔH240R-infected primary porcine alveolar macrophages (PAMs). The ASFV-infected PAMs were fixed and prepared for sectioning at 24 hpi. Sections were incubated with anti-p72 antibodies, followed by incubation with a protein A-gold conjugate (10 nm). The black arrows indicate labeling in the icosahedral capsid, while the red arrows indicate representative labeling observed with antibodies against the capsid proteins in the aberrant structures. (B) Higher magnifications of normal or aberrant structures. The outer electron-dense layers of these structures containing tubules and bilobular structures are indicated by red arrows, while those layers resembling the capsid of a normally assembling particle are indicated by black arrows. (C) The aberrant-to-normal particle ratios of ASFV HLJ/2018 (ASFV-WT) or ASFV-ΔH240R. ASFV-WT- or ASFV-ΔH240R-infected PAMs were examined ultrastructurally at 24 and 48 hpi. Aberrant or normal ASFVs were calculated from 30 infected PAMs. Error bars denote standard errors of the means. The significance of differences between groups (*n *= 3) was determined using Student's *t* test (***, *P < *0.001). (D) Images of intracellular budding of normal or aberrant structures. PAMs were infected with ASFV-WT or ASFV-ΔH240R at 18 hpi. Black arrows point to icosahedral structures reaching the outer envelope. Tubular structures are released by budding (red arrows).

To further determine whether these aberrant structures were abnormal ASFVs, IEM analysis was used to examine the presence in the aberrant structures of p72, which is a marker capsid protein of the normal particles. Anti-p72 antibodies recognized the outermost layer of the icosahedral capsids and the aberrant structures (Fig. 6A and B). The structures without icosahedral morphology containing the ASFV p72 protein were likely to be formed during virus morphogenesis. Actually, as shown in more detail in Fig. 6B, these tubular and bilobulate particles in ASFV-ΔH240R-infected PAMs contained a well-defined and electron-dense outer layer (red arrows) of the normal particles (black arrows), which were constructed in ASFV-WT-infected PAMs. The recombinant virions inducibly expressing the *B438L* gene exhibit a large amount of aberrant tubular structures or bilobulate forms in the absence of the inducer (20). In this study, we found that the tubular and bilobulate structures in ASFV-ΔH240R-infected PAMs were similar to the morphology of pB438L-defective particles. These findings suggest that the formation of normal viral particles is severely hampered in ASFV-ΔH240R during the process of viral morphogenesis.

We further explored the correlation between ASFV-ΔH240R growth defect and aberrant virions. The aberrant-to-normal particle ratio of ASFV-ΔH240R in viral factories was approximately 49:1, remaining constant at 24 and 48 hpi, indicating that this observation did not result from a delay in ASFV-ΔH240R assembly. Overall, the number of tubular structure-containing particles in 30 ASFV-ΔH240R-infected cells (*n *= 360 particles) was 98% that of normal viruses in the 30 cells infected with ASFV-WT (*n *= 375 particles) (Fig. 6C). The results showed that ASFV-ΔH240R exhibited a 2.0-log growth defect in PAMs, of which a 1.7-log growth defect in infectivity is due to the aberrant-to-normal particle ratio (49:1) of ASFV-ΔH240R.

The intracellular transport of MVs from viral factories to the plasma membrane is dependent on the interaction between the protein pE120R and microtubules for efficient dispersion (23, 24). Moreover, these different abnormal structures were released from PAMs by budding through the plasma membrane, which occurred in a manner similar to that of ASFV-WT (Fig. 6D).

Collectively, ultrathin thawed cryosection analyses indicate that the absence of pH240R causes aberrant virion morphogenesis, resulting in decreased icosahedral morphology of intracellular mature particles and increased formation of tubule and bilobulate structures, which reduces infectious progeny virus production, but pH240R is not essential for ASFV transport from the assembly sites to the plasma membrane.

### pH240R is not required for virus attachment to and entry into PAMs.

**FIG 7:**
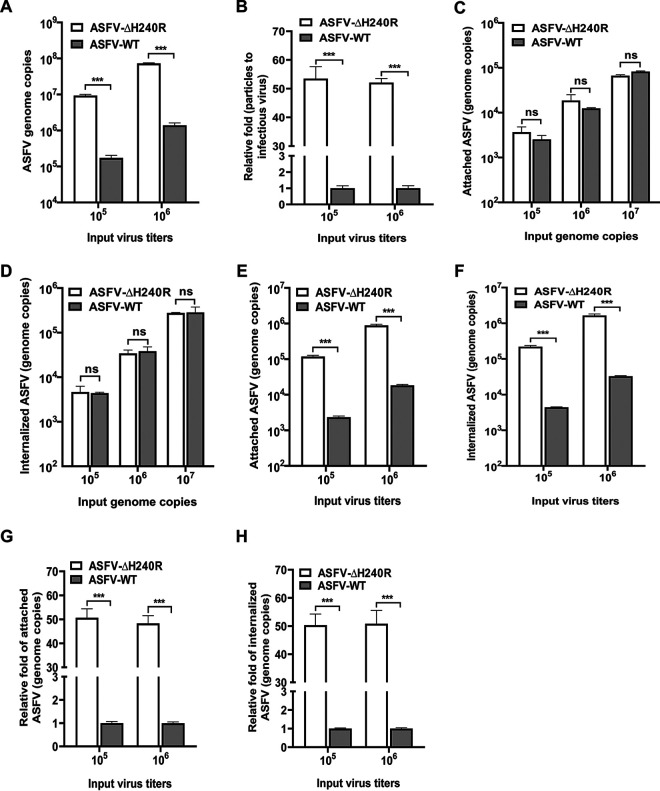
The H240R protein is not required for ASFV binding to or entry into PAMs. (A) Genome copies of ASFV-ΔH240R or ASFV HLJ/2018 (ASFV-WT) per 10^5^ and 10^6^ HAD_50_. Genome copies were determined from 10^5^ and 10^6^ HAD_50_ ASFV-ΔH240R or ASFV-WT by quantitative PCR (qPCR) based on *B646L*. (B) ASFV-ΔH240R or ASFV-WT particle-to-infectious virus ratios. The particle-to-infectious virus ratios of ASFV-ΔH240R are nearly 50 times higher than those of ASFV-WT based on samples from panel A. (C and E) The attachment levels of ASFV-ΔH240R or ASFV-WT were similar. Equal numbers of genome copies (10^5^, 10^6^, and 10^7^) (C) or equal titers (10^5^ and 10^6^) (E) of ASFV-ΔH240R or ASFV-WT were added to PAMs at 4°C and allowed to attach for 1 h. The numbers of genome copies of attached ASFVs were quantified by qPCR. (G) The fold change in attached ASFV-ΔH240R was nearly 50 times higher than that of ASFV-WT with equal titers (E). (D and F) The internalization levels of ASFV-ΔH240R and ASFV-WT were similar. Equal numbers of genome copies (10^5^, 10^6^, and 10^7^) (D) or equal titers (10^5^ and 10^6^) (F) of ASFV-ΔH240R or ASFV-WT were added to PAMs at 37°C and allowed to internalize for 2 h. The genome copies of internalized ASFVs were quantified by qPCR. (H) The fold change in internalized ASFV-ΔH240R was approximately 50 times higher than that of ASFV-WT with equal titers (F). The data shown are from three independent experiments. Error bars denote standard errors of the means. The significance of differences between groups (*n *= 3) was determined using Student's *t* test (***, *P < *0.001). ns, not significant.

To determine whether pH240R is involved in ASFV entry, the binding and entry abilities of ASFV-ΔH240R and ASFV-WT were compared. First, we examined the genome copies of the two viruses with the same titer. As shown in Fig. 7A, the numbers of genome copies of ASFV-ΔH240R were 9.36 × 10^6^ and 7.32 × 10^7^ per 10^5^ and 10^6^ 50% hemadsorption doses (HAD_50_), respectively, while those of ASFV-WT were 1.74 × 10^5^ and 1.41 × 10^6^ per 10^5^ and 10^6^ HAD_50_, respectively, indicating that the numbers of genome copies of ASFV-ΔH240R were 53.5- or 52.11-fold higher than those of ASFV-WT at equal titers (Fig. 7B). The data support the conclusion that the *H240R*-deleted virus produces more viral genome in the supernatants with lower infectivity.

To address whether pH240R is involved in virus attachment and internalization, equivalent amounts of ASFV-ΔH240R and ASFV-WT particles were incubated with PAMs for 2 h at 4°C or 37°C. The attached and endocytosed particles were quantified by detecting the genome copies of *B646L* in PAMs. Comparable amounts of ASFV-ΔH240R and ASFV-WT were attached to or internalized into PAMs when the numbers of input genome copies of the inoculum were equivalent (Fig. 7C and D).

In contrast, when the inoculum contained equal titers of ASFV-ΔH240R and ASFV-WT, a significant difference was found in the numbers of genome copies of *B646L* of virions attached to or internalized into PAMs (Fig. 7E and F), as the genome copies of ASFV-ΔH240R were nearly 50-fold higher than those of ASFV-WT (Fig. 7G and H). The data show that both infectious and noninfectious forms of the *H240R*-deleted virus bind to and are endocytosed into cells, and hence the uptake of viral genome is higher than that of ASFV-WT.

Together, these findings indicate that the defective phenotype of virions produced by ASFV-ΔH240R is not due to a defect in attachment and entry by virions.

### RNA sequencing analysis of differential expression genes in the ASFV-ΔH240R-infected cells.

**FIG 8:**
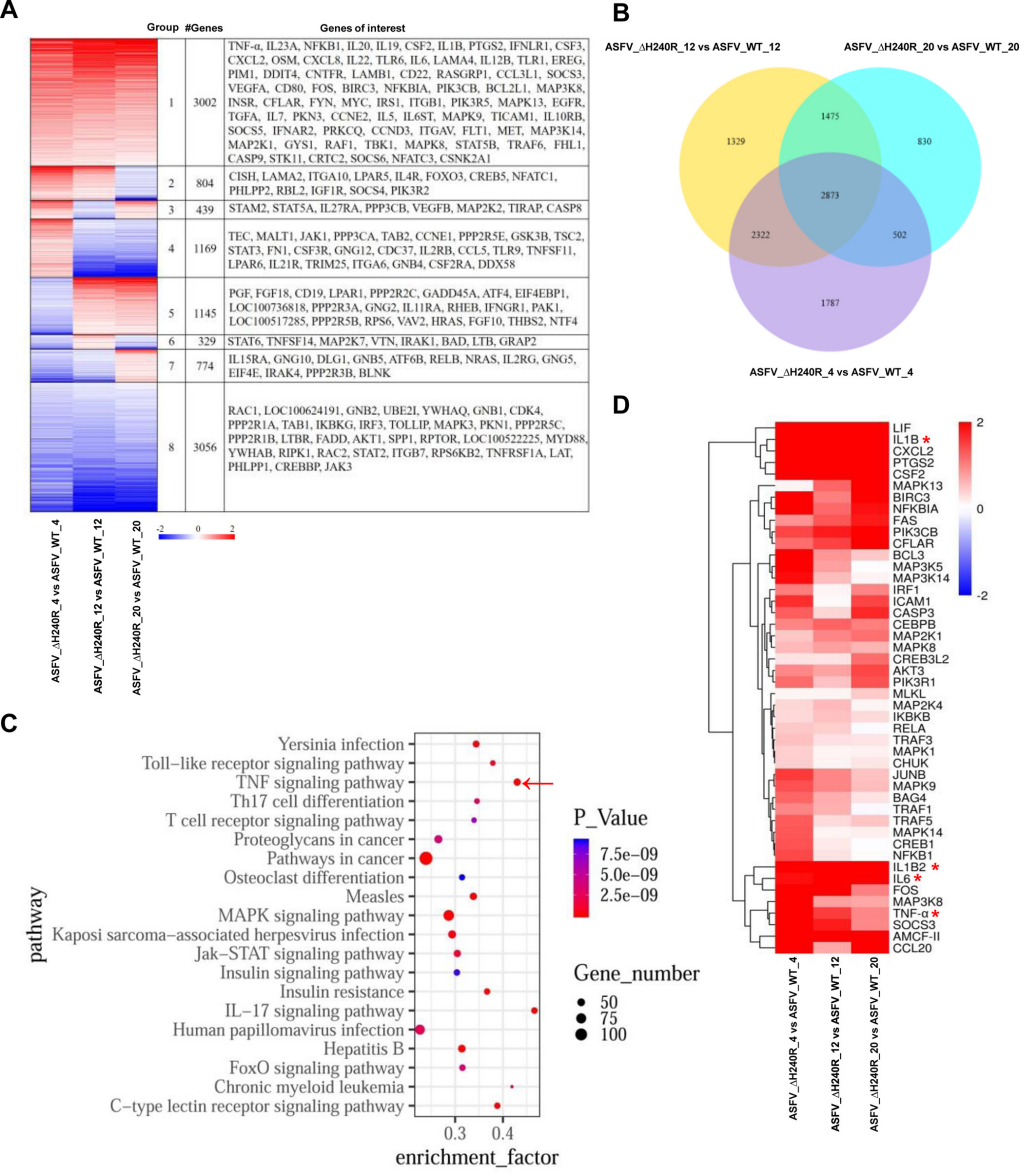
Gene expression profiling in the ASFV-ΔH240R-infected primary porcine alveolar macrophages (PAMs) by RNA-sequencing analysis. (A) Hierarchical clustering analysis of differentially expressed genes (DEGs) in the groups of ASFV-ΔH240R versus ASFV HLJ/2018 (ASFV-WT). PAMs were mock infected or infected with ASFV-ΔH240R or ASFV-WT for 4, 12, and 20 h. Total RNA was extracted from the PAMs and sequenced using the Illumina HiSeq 2500 platform. Each group of DEGs and genes of interest is shown on the right. (B) Venn diagrams of mRNAs in different groups. The overlapping section represents the number of common mRNAs in each group, while the nonoverlapping sections represent the numbers of group-specific mRNAs. (C) Top 20 pathways from KEGG enrichment analysis of 3,002 upregulated genes. The TNF signaling pathway is indicated by a red arrow. (D) Analysis of the 46 upregulated genes in the TNF signaling pathway induced by ASFV-ΔH240R versus ASFV-WT in the three groups. Asterisks indicate upregulated expression of target genes.

To investigate the host immune responses of PAMs induced by infection with ASFV-ΔH240R compared with ASFV-WT, RNA sequencing (RNA-seq) analysis was used to characterize differentially expressed genes (DEGs). A total of 10,718 DEGs were clustered into 8 clusters with unique expression patterns (Fig. 8A). We were interested in cluster 1, with 3,002 genes, of which expression remained transcriptionally upregulated throughout the three time points, including TNF-α, IL-1β, CXCL8, IL-6, IL-10, and others associated with the inflammatory response. Clusters 2, 3, and 4 had 2,412 genes, which were first significantly upregulated and then decreased to lower levels. Clusters 5, 6, and 7 had 2,248 genes, which were first greatly downregulated and then increased to higher levels. The 3,056 genes in cluster 8 were downregulated throughout the time course. The expression of 7,484, 7,999, and 5,680 genes was significantly altered following ASFV-ΔH240R infection compared with ASFV-WT at 4, 12, and 20 hpi (with a fold change of >2; *P < *0.05), and a total of 2,873 overlapping DEGs were identified at different time points (Fig. 8B). However, the functions of these upregulated genes remain unknown. So the pathways associated with these upregulated genes were analyzed with the Kyoto Encyclopedia of Genes and Genomes (KEGG) database. The top 20 enriched pathways of 3,002 upregulated genes at all three time points are shown in Fig. 8C. In comparison with the ASFV-WT-infected PAMs, the majority of the dysregulated mRNAs during ASFV-ΔH240R infection were involved in innate immunity signaling (including TNF, MAPK, and JAK-STAT signaling pathways), receptor signaling, metabolic networks, and other pathways involved in viral infection. The 46 upregulated genes were significantly enriched in the TNF signaling pathway (red arrow). The altered cellular mRNAs from the TNF signaling pathway are shown in Fig. 8D; IL-1β, TNF-α, and IL-6 were highly transcribed during ASFV-ΔH240R infection compared with ASFV-WT at 4, 12, and 20 hpi.

### ASFV-ΔH240R induces higher inflammatory cytokine production in PAMs than does ASFV-WT.

**FIG 9:**
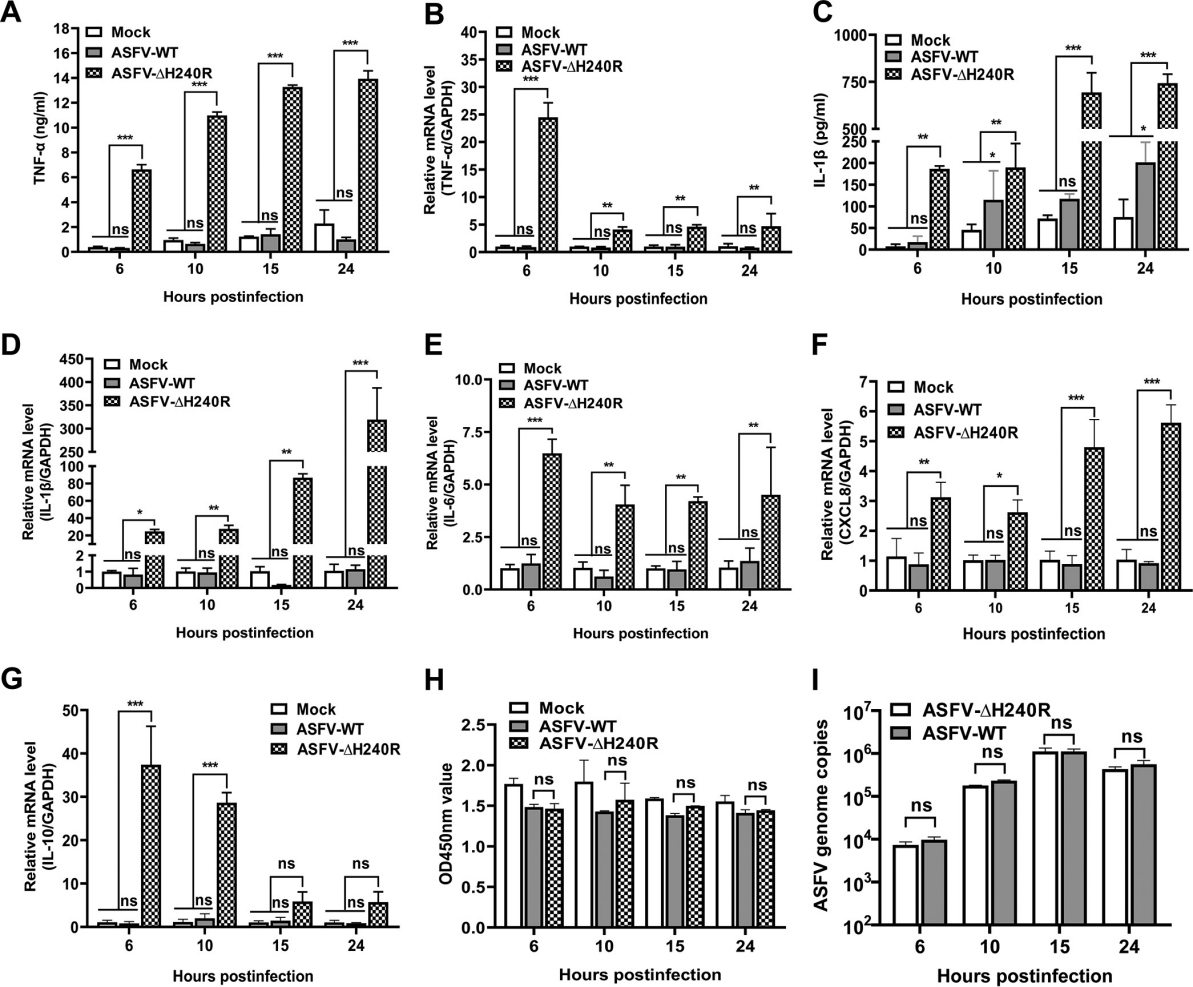
ASFV-ΔH240R induces higher cytokine production than ASFV-WT. (A to G) Primary porcine alveolar macrophages (PAMs) were either mock infected or infected with ASFV-ΔH240R or ASFV HLJ/2018 (ASFV-WT) at equal numbers of genome copies of 10^7^. At 6, 10, 15, or 24 hpi, the expression levels of TNF-α (A) and IL-1β (C) in the cell culture supernatants were detected by enzyme-linked immunosorbent assay kits, and the mRNA levels of TNF-α (B), IL-1β (D), IL-6 (E), CXCL8 (F), and IL-10 (G) in the cell lysates were determined by quantitative RT-PCR. (H) Cell viability was analyzed using a CCK-8 counting kit. (I) The genome copies of ASFV were measured by quantitative PCR. Error bars denote standard errors of the means. The significance of differences between groups (*n *= 3) was determined using Student's *t* test (*, *P < *0.05; **, *P < *0.01; ***, *P < *0.001). ns, not significant.

To validate the upregulated DEGs associated with the inflammatory response from transcriptional data upon viral infection, PAMs were infected with ASFV-ΔH240R or ASFV-WT at the same number of genome copies of 10^7^ for 6, 10, 15, and 24 h, and the secretion and mRNA expression of several cytokines were detected by enzyme-linked immunosorbent assay (ELISA) and qRT-PCR. As a result, compared with ASFV-WT, ASFV-ΔH240R induced much higher levels of TNF-α and IL-1β and the downstream cytokines IL-6, CXCL8, and IL-10. TNF-α secretion induced by ASFV-ΔH240R was approximately 10-fold higher than that induced by ASFV-WT at each time point (Fig. 9A), and TNF-α mRNA expression was approximately 25-fold higher at 6 hpi in PAMs (Fig. 9B). IL-1β secretion induced by ASFV-ΔH240R infection was approximately 4-fold higher than that of ASFV-WT at each time point (Fig. 9C), and IL-1β mRNA expression was approximately 300-fold higher at 24 hpi in PAMs (Fig. 9D). IL-6, CXCL8, and IL-10 mRNA expression induced by ASFV-ΔH240R infection was increased by approximately 5-, 5-, and 10-fold, respectively, compared with ASFV-WT (Fig. 9E to G). To exclude the possibility that the enhanced induction of cytokines by ASFV-ΔH240R was due to the increased cell viability or more virus particles, we detected the cellular viabilities and viral genome copies following viral infections. The results showed that the cellular viability of PAMs was not obviously different following infection with ASFV-ΔH240R or ASFV-WT (Fig. 9H), and the number of genome copies of ASFV-ΔH240R was similar to that of ASFV-WT (Fig. 9I). Taken together, our results reveal that infection with ASFV the *H240R*-deleted ASFV mutant activates inflammatory signaling pathways in comparison with ASFV-WT infection.

## DISCUSSION

Of more than 150 ORFs (8) encoded by the ASFV genome, only a few have been elucidated in detail (25–27). Dissecting the functional roles of viral proteins in ASFV is crucial for understanding the process of virus replication *in vitro* as well as the virulence of ASFV *in vivo*, which will accelerate the development of novel ASF vaccines and antivirals. According to a previous report, more than 50 structural proteins are assembled into mature ASFV particles (10). It is well accepted that viral structural proteins generally play important roles in virus entry, assembly, and virulence. Identification of the functions of virus structural proteins, followed by their genetic manipulation, has enabled the development of potential attenuated ASFV vaccine candidates (19, 20, 24, 25, 28–36). A recent study revealed that the ASFV capsid structure is built from 17,280 proteins, including one major and four minor capsid proteins (18). As shown previously, morphogenesis of the icosahedral capsid in the process of ASFV assembly requires the proteins p72 and pB438L (19, 20). Here, we have shown that pH240R is highly conserved among ASFV isolates and encodes a late viral structural protein that modulates viral growth by affecting virion morphogenesis in PAMs.

In fact, there is a lack of significant similarity between ASFV pH240R and other viral or nonviral proteins available in the databases. By using RNA transcriptional kinetics combined with IEM, pH240R was shown to be a late structural protein (Fig. 1B) that was located in the icosahedral capsid of the virus particle (Fig. 2C). As a capsid protein, pH240R may be involved in ASFV morphogenesis by interacting with viral inner membrane and capsid proteins. Remarkably, we showed that pH240R interacted with the MCP p72 (Fig. 2D to F), indicating that pH240R plays an important role in the formation of the icosahedral capsid. Since viral proteins exert multiple functions, we cannot rule out the possibility that pH240R interacts with other viral proteins in other stages of the ASFV life cycle.

In this study, we found that ASFV-ΔH240R was approximately 2.0 logs less infectious than ASFV-WT (Fig. 3D and E), as deduced from multistep and one-step virus growth curves. Although ASFV-ΔH240R binds efficiently to the cell surface and is endocytosed and subsequently disassembled (Fig. 7C to H), similar to ASFV-WT, a more significant phenotype of ASFV-ΔH240R was the contribution of pH240R to the morphogenesis of viral icosahedral particles. Most of our knowledge on ASFV morphogenesis derives from TEM analysis of the morphological stages of virus assembly (12, 13, 17, 30, 37, 38) and the localization of virus proteins on the virus precursors and MVs (9, 13, 18, 39–41). pB119L affected the maturation of normal virions, virions containing acentric nucleoid structures (42). In this study, in the absence of pH240R, few viral icosahedral particles were observed within the infected PAMs by TEM analysis. In contrast, large aberrant tubular structures of viral origin, as well as bilobulate forms, were generated (Fig. 5A and B). The morphology of ASFV with the *H240R* deletion was similar to that of virus with the *B438L* deletion (20). In the case of icosahedral viruses, it has been shown that structures of *Sericesthis* iridescent virus with tubular morphology arise from the loss of pentameric capsomers (43). We presumed that the incorrect assembly of ASFV-ΔH240R is related to a defect in the external structure, the capsid. Indeed, this capsid is to some extent functional, as the filamentous structures can move from the virus assembly sites to the plasma membrane and exit cells by budding. The other anomalous structures generated in ASFV-ΔH240R-infected PAMs, the bilobulate forms, present the domain of a developing normal particle. Importantly, the morphological similarity observed in the tubular and bilobulate forms (Fig. 6B), together with the fact that the diameter of the lobules is the same as that of the tubules, suggests a morphogenetic relationship between the two structures. However, we did not obtain images of these bilobulate structures translocated by budding to the plasma membrane.

Our data suggest that the 2.0-log growth defect in ASFV-ΔH240R-infected PAMs is partially attributable to a reduction in the infectivity of the actually produced abnormal ASFV particles with an overall ratio of 49:1 (aberrant to normal particles) (Fig. 6C). The data showed a 1.7-log reduction in ASFV-ΔH240R infectivity due to the noninfectious abnormal ASFV particles, meaning that only 1 out of 50 ASFV-ΔH240R virions were normal and infectious. Overall, the reduction in ASFV-ΔH240R infectivity was mainly due to the production of noninfectious virions with aberrant tubular and bilobulate structures. Since ASFV-ΔH240R exhibited decreased replication efficiency in PAMs, we assumed that the ASFV mutant might not replicate well *in vivo*. Further investigation is needed to support this speculation.

A previous study has shown that p72 is the MCP and constitutes most of the external capsid, whereas the penton complex forms the vertex of the icosahedral capsid (9, 18). The penton and minor capsid proteins form a complicated network under the outer capsid shell to stabilize the whole capsid (9, 18). In the absence of pH240R, the generated viral particles possess a tubular structure in which most icosahedral symmetry is lost, which suggests that the formation of capsid pentasymmetrons may be compromised under these circumstances. Using IEM analysis, we were able to show that pH240R is a capsid component of ASFV virions. It is possible that loss of icosahedral symmetry supports a role for the protein in the construction or stabilization of the icosahedral capsid of the virus particle in the absence of pH240R. In the ASFV life cycle, viral capsids are assembled in viral factories, and host membrane proteins are incorporated into virions during virus egress (10). The capsid structures have been solved by cryo-EM analysis in previous studies (9, 18, 44), but outer envelope proteins remain to be dissected. It is well accepted that virion morphology is determined on the structure of the capsid. Considering that the morphology of the majority of mature virions was changed upon the *H240R* deletion, it is possible that the composition of outer membrane proteins cannot be affected.

ASFV encodes at least 16 proteins involved in the assembly of virus particles, as previously described (9). The stability of the capsid is determined by the penton complex, which is composed of five subunits (18). In this study, few icosahedral virions could still be observed in ASFV-ΔH240R-infected PAMs (Fig. 5A and B). It is likely that the function of pH240R could be compensated for by other penton proteins encoded by ASFV, but other penton proteins alone are not sufficient to completely compensate for pH240R to prompt all virions to be assembled into normal icosahedral structures, because other penton proteins still exert their own functions.

The innate immune response is the first line of host defense against viral infection (45). Previous studies demonstrated that inflammatory cytokines are key components of the host innate immunity to inhibit viral replication, such as adenovirus (46) and West Nile virus (47), and clear infected immune cells by inflammatory cell death. However, ASFV infection induced low-level inflammatory cytokines, as previously described (32). In this study, virus growth was reduced and high-level inflammatory cytokines were induced by ASFV-ΔH240R infection. We speculated that the partial growth defect in ASFV-ΔH240R infection may be due to the activation of the inflammatory signaling pathway to block the virus replication in PAMs. In other words, the production of cytokines, including IL-1β, TNF-α, IL-6, CXCL8, and IL-10, inhibited the replication of ASFV-ΔH240R (Fig. 9). ASFV-ΔH240R infection produced noninfectious progeny virions, leading to decreased growth, and at the same time induced inflammatory cytokine expression, which further inhibited the replication of the progeny virus. Actually, the two aspects are a complex process that negatively regulates viral replication. However, the mechanism by which pH240R is involved in the inflammatory signaling pathway requires further investigation.

In summary, this study represents the first report that pH240R is essential for ASFV icosahedral capsid formation and infectious particle production and affects inflammatory cytokine expression in PAMs. Our study not only deepens our understanding of the function of pH240R in the ASFV life cycle but also provides a new target for vaccine and drug development to prevent and control this disease, which is threatening the global pork industry.

## MATERIALS AND METHODS

### Cells, viruses, and antibodies.

PAMs (22) and HEK293T cell lines, which were derived from our own stock stored in liquid nitrogen, were cultured with RPMI 1640 medium (catalog no. C11875500BT; Gibco) supplemented with 10% heat-inactivated fetal bovine serum (FBS) (catalog no. 10099-141C; Gibco) and 4% antibiotics-antimycotics (10,000 IU/mL penicillin, 10,000 μg/mL streptomycin, and 25 μg/mL amphotericin B) (catalog no. 15240–062; Gibco) in a 37°C incubator with 5% CO_2_. The ASFV Pig/Heilongjiang/2018 (ASFV HLJ/2018) strain was isolated from field samples in China as described previously (GenBank accession no. MK333180.1) (48). ASFV-P31, a cell-adapted ASFV strain, replicates efficiently in HEK293T cells (22).

A rabbit anti-Flag polyclonal antibody (PAb) (catalog no. ab1162; Sigma-Aldrich), rabbit anti-Myc PAb (catalog no. ab9106; Sigma-Aldrich), and mouse anti-Flag monoclonal antibody (MAb) (catalog no. ab62928; Sigma-Aldrich) were purchased from Sigma. A mouse anti-PPA IBC11 (anti-p72) MAb (catalog no. 160620; Ingenasa) was purchased from Ingenasa. A homemade rabbit anti-H240R PAb was raised against the recombinant H240R protein of ASFV-WT. Alexa Fluor 488-conjugated goat anti-mouse IgG (H+L) (catalog no. 1942237; Invitrogen), Alexa Fluor 488-conjugated goat anti-rabbit IgG (H+L) (catalog no. 2072687; Invitrogen), Alexa Fluor 568-conjugated goat anti-mouse IgG (H+L) (catalog no. 175697; Invitrogen), and Alexa Fluor 594-conjugated goat anti-rabbit IgG (H+L) (catalog no. 111-585-003; Invitrogen) antibodies were purchased from Invitrogen. 4′,6-Diamidino-2-phenylindole (DAPI) (catalog no. C006; Solarbio) and a mouse anti-GST MAb (catalog no. K200006M; Solarbio) were purchased from Solarbio.

### Construction of plasmids.

The *H240R* gene was cloned into the pCMV-Myc (Clontech) and pGEX-6P-1 (GE Healthcare) vectors to generate pMyc-pH240R and pGST-pH240R, respectively. The *D117L*, *E248R*, *CP312R*, *B438L*, and *B646L* genes were cloned into the pCAGGS-Flag vector (Clontech) to obtain pFlag-p17, pFlag-pE248R, pFlag-p*C*P312R, pFlag-pB438L, and pFlag-p72. The primers used in the study are shown in Table 1.

### Detection of *H240R* gene transcription in ASFV-infected PAMs.

To dissect the transcriptional kinetics of the *H240R* gene, monolayers of PAMs prepared in 24-well plates were infected with ASFV-WT at an MOI of 5 and incubated for 1 h at 37°C with 5% CO_2_ to allow virus attachment. Then, the supernatants were removed and replaced with fresh medium. The infected PAMs were incubated for 1, 4, 7, 10, or 24 h at 37°C with 5% CO_2_. The total RNA was extracted from the PAMs as recommended by the manufacturer’s protocols using RNAiso Plus (catalog no. 9109; TaKaRa). RNA was treated with DNase (catalog no. 143582; Roche) to remove genome DNA. The cDNA was processed in a 20-μL volume with avian myeloblastosis virus (AMV) reverse transcriptase XL (catalog no. 2621; TaKaRa) and used as a template for qRT-PCR. The *B646L* cDNA was quantified by qRT-PCR in a reaction mixture containing 10 μM p72 forward and reverse primers (Table 1) and 2 μL of the cDNA template under the condition of 1 min at 95°C, followed by 40 cycles of 95°C for 30 s, 55°C for 30 s, and 72°C for 30 s. Under the same conditions, the *CP204L* and *H240R* cDNAs were quantified in a single reaction mixture containing 10 μM forward and reverse primers (Table 1). An internal *GAPDH* control was used in the experiment.

### GST pulldown assay.

GST pulldown was performed as described previously (49). Briefly, purified GST-pH240R or GST protein was incubated with lysates from the cells transfected with pFlag-p17, pFlag-pCP312R, pFlag-pE248R, pFlag-pB438L, and pFlag-p72 at 4°C for 12 h, respectively. The proteins pulled down by glutathione-Sepharose 4B resin (catalog no. 17-0756-01; GE Healthcare) were examined by Western blotting using a rabbit anti-Flag MAb and a mouse anti-GST MAb.

### Laser confocal microscopy.

For the analysis of subcellular localization of pH240R, HEK293T cells on laser confocal microscopy plates were transfected with pMyc-pH240R or pCMV-pMyc for 24 h. For the colocalization assay of p72 and pH240R, HEK293T cells were cotransfected with pMyc-pH240R and pFlag-p72 for 24 h; HEK293T cell monolayers were infected with ASFV-P31 at an MOI of 1 for 48 h. Then, the cells were incubated with the relevant antibodies and examined with a Zeiss LSM 880 laser-scanning confocal microscope as described previously (50).

### Co-IP assay.

HEK293T cells were infected with ASFV-P31 at an MOI of 1 for the co-IP assay. At 48 hpi, the cells were lysed with NP-40 for 30 min at 4°C and centrifuged for 10 min at 4°C; the cellular supernatants were incubated with an irrelevant isotype antibody IgG (catalog no. A7016; Beyotime) serving as a control and anti-H240R PAb with protein G plus agarose (catalog no. IP10-10ML; Merck-Millipore) at 4°C for 12 h. The agarose was washed six times with phosphate-buffered saline (PBS), and the bound proteins were subjected to Western blotting (49).

### Generation and identification of ASFV-ΔH240R.

A recombinant transfer vector, pOK12-p72EGFP-ΔH240R, was constructed as described previously (51). pOK12-p72EGFP-ΔH240R containing genomic positions flanking the targeted gene mapping approximately 1 kb to upstream and downstream homologous arms and a reporter gene cassette containing the EGFP reporter with the ASFV p72 late gene promoter were used. The left and right arms flanking the target gene are located in the ASFV-WT genome at positions 154139 to 155338 and 156065 to 157264, respectively. Nucleotides in the genome at positions 155339 to 156064 are replaced by the expression cassette containing the EGFP reporter. Briefly, the left and right arms were amplified by PCR and assembled to contain the EGFP reporter harboring restriction enzyme sites at both termini by overlapping PCR. The cassette was then cloned into the linearized pOK12 vector to generate the recombinant transfer vector pOK12-p72EGFP-ΔH240R using the Vazyme ClonExpress II one step cloning kit (Vazyme Biotech Co., Ltd., China).

The recombinant ASFV-ΔH240R was generated by homologous recombination between the parental virus ASFV (ASFV-WT) genome and the recombination transfer vector by infection and transfection procedures in PAMs (51). The construction generated an expected gene deletion in the *H240R* ORF. The resulting virus from the homologous recombination event was purified by successive limiting dilution on PAM monolayers. The purified ASFV-ΔH240R was amplified in PAMs to make a virus stock. To ensure the absence of the desired deletion in each recombinant genome, viral DNA was extracted from ASFV-ΔH240R-infected PAMs and identified by PCR using specific primers targeting these genes (Table 1) and sequencing analysis.

### Next-generation sequencing of the ASFV-ΔH240R genome.

PAMs were seeded in 6-well plates and infected with ASFV-ΔH240R, from which DNA was extracted as described above. Full-length sequencing of the whole genome was performed using the Illumina HiSeq 2500 platform of Novogene Technology Co., Ltd. (Beijing, China). Among the total 20 million reads, 250,000 reads matching the genome were obtained. The high-quality reads were mapped to the genome of ASFV-WT (GenBank accession no. 6E4B and MN715134.1) using Hisat2.

### Determination of ASFV-ΔH240R replication kinetics.

Comparative growth curve assays between ASFV-ΔH240R and ASFV-WT were performed in PAMs. PAM monolayers were prepared in 24-well plates and infected with the viruses at an MOI of 0.01 or 3. After 1 h of adsorption, the cells were rinsed twice with PBS. The monolayers were then incubated with medium at an MOI of 0.01 for 2, 24, 48, 72, 96, and 120 h and incubated at an MOI of 3 for 2, 12, 16, 20, 24, and 36 h. At different time points, the ASFV-infected cultures were stored at −70°C. Subsequently, the thawed lysates were used to determine viral titers in HAD_50_/mL in PAMs.

### Hemadsorption assay.

Since the CD2v protein mediates the hemadsorption of ASFV around virus-infected cells in the presence of porcine red blood cells, a hemadsorption assay was used to evaluate the median tissue culture infectivity of ASFV (52, 53). Around 5 × 10^4^ PAMs seeded in 96-well plates were infected with ASFV-ΔH240R or ASFV-WT for 48 h. Then, the cells were incubated with 5 × 10^5^ porcine red blood cells diluted in PBS, and hemadsorption was observed on the fifth day.

### qPCR assay.

ASFV genomic DNA was extracted from the cells or cell supernatants using the MagaBio plus virus DNA purification kit (catalog no. 9109; BioFlux) according to the manufacturer’s protocols. ASFV genomic DNA copies were quantified by qPCR on the QuantStudio system (Applied Biosystems, USA) based on a previously described method (24).

### Virus genome replication kinetics analysis.

The ASFV genome replication assay was conducted in 24-well plates. PAMs grown in 24-well plates were infected with the viruses at genome copies of 10^7^. After 1 h of virus adsorption, the inoculum was removed, and PAMs were rinsed twice with PBS. The cells were then incubated with medium for 2, 6, 12, 18, 24, and 30 h at 37°C with 5% CO_2_. The intracellular or extracellular ASFV genomic DNA was quantitated by qPCR as described above. All samples were examined in triplicate.

### Virus adsorption and entry assays.

ASFV adsorption and entry assays were conducted in PAMs grown in 24-well plates. For the binding assay, cells were infected with equal numbers of genome copies (10^5^, 10^6^, and 10^7^) or equal titers (10^5^ and 10^6^) of virions for 2 h at 4°C. After washing five times with PBS, the cells were lysed in buffer for DNA extraction and examination of ASFV genome copies. For the internalization assay, PAMs were infected with equal numbers of genome copies (10^5^, 10^6^, and 10^7^) or equal titers (10^5^ and 10^6^) of virions at 37°C for 2 h. After five cycles of washing, to remove surface-bound virus, the cells were treated with 0.05% trypsin (catalog no. 25300-054; Gibco) for 5 min and proteinase K (catalog no. 539480; Merck) for 2 min. After three additional washes, the cells were lysed in buffer for DNA extraction and examination of ASFV genome copies. All samples were analyzed in triplicate.

### TEM IEM.

For virus assembly assay, PAMs seeded on 60-mm-diameter plates were infected with ASFV-WT or ASFV-ΔH240R at an MOI of 5 and fixed with 2% glutaraldehyde in PBS for 1 h at 18 hpi. The samples were dehydrated with acetone and embedded in epoxy according to standard procedures. After polymerization, 80-nm-thick (ultrathin) sections were obtained and stained with uranyl acetate and lead citrate according to standard procedures. The samples were analyzed on an H-7650 (Hitachi, Tokyo, Japan) operated at 80 kV.

For IEM analysis, cells were fixed at the indicated times with 4% paraformaldehyde and 1% glutaraldehyde in 0.1 M HEPES at pH 7.2 for 2 h at 4°C. The fixative was then removed, and gradient dehydration was performed with 50%, 70%, 90%, and 100% dimethylformamide (DMF) for 15 min at 4°C, respectively. The cells were then embedded in LR white resin and polymerized for 10 days under UV irradiation at −20°C. Subsequently, the fixed cells were prepared for sectioning and immunolabeling as described previously (44).

### RNA-seq analysis.

For the global transcriptomic analysis, PAMs infected with 10^7^ genome copies of ASFV-ΔH240 or ASFV-WT were used for extraction of RNA at 4, 12, and 20 hpi using RNAiso Plus reagent. All the sample analyses were carried out in triplicate. Genome-wide differential gene expression analyses were performed using RNA deep sequencing by Novogene Technology Co., Ltd. (Beijing, China). Poly(A) plus RNAs were sequenced using the Illumina HiSeq 2500 platform. The high-quality reads were mapped to the reference porcine genome using Hisat2 (https://daehwankimlab.github.io/hisat2/), and the reference-based assembly of transcripts was performed using Stringtie (http://ccb.jhu.edu/software/stringtie/). Kyoto Encyclopedia of Genes and Genomes (KEGG) enrichment analysis and bioinformatics analysis were performed to analyze the generated RNA-seq data.

### Detection of inflammatory cytokines.

Approximately 10^6^ PAMs were infected with 10^7^ genome copies of ASFV-ΔH240 or ASFV-WT for 1 h at 37°C and then rinsed twice with PBS. PAMs were then cultured in new medium for 6, 10, 15, and 24 h. The cell supernatants were collected to measure the concentrations of several cytokines by ELISA kits (Ray Biotech, Norcross, GA). The cells were collected for qRT-PCR analysis, and uninfected cells were used as a negative control. All samples were analyzed in triplicate.

### Statistical analysis.

Statistical analyses were performed using SPSS 22.0 software (SPSS Software, Inc.). Differences between groups were examined for statistical significance using Student's *t* test. An unadjusted *P* value of less than 0.05 was considered significant.
